# Classroom Placement and Twins’ Social Behaviors in Elementary School: Providing Empirical Evidence to Inform Educational Policy

**DOI:** 10.1177/08959048211015626

**Published:** 2021-05-27

**Authors:** Gabrielle Garon-Carrier, Vincent Bégin, Mara Brendgen, Frank Vitaro, Isabelle Ouellet-Morin, Ginette Dionne, Michel Boivin

**Affiliations:** 1 Université de Sherbrooke, Sherbrooke, QC, Canada; 2 Université de Montréal, Montreal, QC, Canada; 3 Université du Québec à Montréal, Montreal, QC, Canada; 4 Université Laval, Quebec, QC, Canada

**Keywords:** classroom placement, twins’ similarity, social behaviors, intertwin relationship, educational policy

## Abstract

Classroom placement of twins is an ongoing issue for educational policy. Many educational jurisdictions have standard policy most commonly founded in the belief that separation supports individual identity, personal development and academic opportunity. This study examined the effects of classroom placement in a sample of 560 twin pairs whose behaviors were assessed from ages 5 to 12 years. We found no detrimental effect of classroom sharing on twins’ social development. In contrast, this study provides evidence that educating twins together is associated with modest positive twins’ behaviors and social functioning at school. Implications for educational policies are further discussed.

When twins start elementary school, parents frequently wonder whether classroom sharing or separation would be beneficial for their social adjustment at school (Segal & Russell, 1992; Staton et al., 2012). It has been suggested that classroom separation favors twins’ individual identities (Alexander, 2012; DiLalla, 2006; Hay & Preedy, 2006; Gordon, 2015; Lalonde & Moisan, 2003; Staton et al., 2012). When twins are assigned to different classrooms, classmates can get to know them as individuals rather than as a pair. Classroom separation has also been proposed to reduce intertwin competition, especially if one of the twins struggles academically (Alexander, 2012; Hay & Preedy, 2006). Indeed, one reason invoked for classroom separation at school entry is to reduce potential risks of a conflictual intertwin relationship (Staton et al., 2012), and reduced social development and relationships beyond the co-twin.

Other arguments have ruled in favor of sharing the same classroom. Given the special bond that unites co-twins, parents sometimes assume that classroom separation in early grades will increase the risk for emotional and behavioral maladjustment (Alexander, 2012; Hay & Preedy, 2006; Lalonde & Moisan, 2003; Staton et al., 2012). School entry is a stressful experience for most children (Parent et al., 2019), and could thus generate further anxiety during transition to formal schooling if co-twins are separated for the first time of their life. Some twins may also negatively respond to forced separation with feelings of sadness, anger, fright and loneliness (Grime, 2008). One additional argument for classroom sharing is that separating twins could unnecessarily distract them (e.g., one twin wondering what his/her co-twin is doing in the next classroom) and increase their inattention behaviors (Grime, 2008), which could hamper their learning at school.

## Classroom Placement Policy

As stated by the Declaration of Rights and Statement of Needs of Multiple Birth children (Council of Multiple Birth Organizations of the International Society for Twin Studies, 1995), education and public awareness regarding multiple birth’s specific developmental processes are needed in order to reduce discrimination and inflexible policies which fail to accommodate their needs. One literature review revealed that many schools implement strict guidelines for classroom placement of twins, despite the fact that impacts of such policies on twins’ development and educational attainment are unknown (Beauchamp & Brooks, 2003). In US, policies are often at the state or district level. Individual schools have the authority to define specific guidelines and policies, depending on the school principal’s point of view (Gordon, 2015; Nilsson et al., 2010). However, in some US states, parents’ right to decide the classroom placement of their twins is supported by law. Official Canadian guidelines for classroom placement of multiple births also recommend leaving the final decision to parents, teachers, principals, and at some ages, children themselves, jointly. Multiple Birth Canada suggests that classroom placement for multiples be evaluated each year in order to ascertain which is the best situation for them (i.e., being together or separated). Despite these recommendations, separation of the co-twins is often encouraged by the School Commission Boards (Lalonde & Moisan, 2003), without considering the costs and benefits of separation, the twins’ own preference, or the individual variation in effects of classroom separation.

A survey conducted among 131 US elementary schools reported that 71.0% of school principals believed that twins should be separated in kindergarten (Gordon, 2015). Those school principals claimed that twins are doing better academically when separated, and that classroom separation is necessary to promote social independence between the twins (Gordon, 2015). However, this notion is not supported by twin studies that examined the effects of classroom placement during the elementary school years, whereby no substantial differences have been associated with classroom placement regarding the twins’ academic achievement (Polderman et al., 2010; White et al., 2018), cognitive abilities (Byrne et al., 2010; Coventry et al., 2009; Grasby et al., 2019; Kovas et al., 2007), and school motivation (Kovas et al., 2015; White et al., 2018). Nonetheless, it is not yet clear whether classroom placement is associated with twins’ social behaviors at school or if classroom separation enhances the twin’s differences in this regard. In fact, only a handful of studies have examined whether classroom placement contributes to twins’ social and behavioral adjustment.

## Average Classroom Placement Effect on Twins’ Behaviors

One UK twin study investigated this question on 878 same-sex twin pairs from the Environmental Risk (E-risk) Longitudinal Twin Study (Tully et al., 2004). Teachers rated twins’ symptoms of attention-deficit and hyperactivity disorder, internalizing and externalizing behaviors, and prosocial behaviors at ages 5 and 7. In comparison to twins sharing the same classroom, twins in separate classrooms had higher scores of internalizing behaviors at age 5. These differences in internalizing behaviors persisted at age 7 for monozygotic (MZ) twins, but not for dizygotic (DZ) twins. It was, however, unknown whether the MZ twins already had higher levels of internalizing behaviors prior to formal schooling. No significant mean differences were noted for symptoms of attention-deficit and hyperactivity disorder, externalizing and prosocial behaviors by classroom placement.

Similar results were reported in another study that statistically controlled for initial differences in functioning before school entry (van Leeuwen et al., 2005). The authors examined the associations of classroom placement at age 5 with externalizing (aggressive and rule breaking behaviors) and internalizing (anxious/depressed, somatic complaints and withdrawn) behaviors at age 7. They also tested whether the most frequent type of classroom placement used during the elementary school years was associated with the externalizing and internalizing behaviors at age 12. This was investigated in more than 7,000 Dutch twin pairs from the Netherlands Twin Register. Controlling for externalizing and internalizing behaviors at age 3, separated twins at age 5 had higher levels of internalizing behaviors at age 7 than twins sharing the same classroom (effect size of 0.14 standard deviation) (van Leeuwen et al., 2005). No such difference was found in levels of externalizing behaviors at age 7 or in levels of internalizing and externalizing behaviors at age 12, suggesting a transient effect on social behaviors.

Another UK twin study examined the contribution of classroom placement at age 7 on externalizing (conduct problems, hyperactivity, peer problems) and internalizing (anxiety) behaviors among 1,941 MZ twin pairs from the Twins Early Development Study (DiLalla & Mullineaux, 2008). Linear regression models were conducted with one twin randomly selected from each pair. Controlling for sex and parent ratings of twins’ behaviors at age 4, results revealed that sharing the same classroom was associated with fewer peer problems at age 7 (DiLalla & Mullineaux, 2008). Classroom placement, however, only explained 3.0% of the variance in peer problems, and it did not significantly predict twins’ levels of conduct problems, hyperactivity or anxiety.

## Similarity Among Twin Pairs Sharing the Same Classroom

Although classroom separation has been argued to favor twins’ social independence and individual identities, very few studies tested whether twins sharing the same classroom displayed more similar social behaviors than twins in different classrooms. One study found twins in the same classroom to be more similar with regard to their internalizing behaviors than twins in different classrooms at age 7. This effect was not due to preexisting differences, suggesting that classroom sharing contributed to twins’ higher similarity (van Leeuwen et al., 2005). Another study using MZ within-pair difference scores also found that being placed in separate classrooms was associated with twins’ dissimilarity in self-reported behaviors and social experiences such as peer victimization or the number of friends (Asbury et al., 2017).

Additionally, higher intraclass (within-pair) correlations were found for teacher reports of internalizing and externalizing behaviors among both MZ and DZ twin pairs taught together at ages 7, 10 and 12 (de Zeeuw et al., 2015; Lamb et al., 2012). Intraclass correlations of hyperactivity, opposition, and ADHD could not be equated between twins sharing the same classroom and twins in separate classrooms (de Zeeuw et al., 2015). A lower estimate of heritability of these externalizing behaviors was found for twins in separate classrooms, leading to a presumed larger contribution of the unique (or unshared) environment, as distinct factors may elicit these behaviors between different classroom environments. Children taught by different teachers, with different rules and teaching methods, and with different peers, could then manifest more dissimilar social behaviors as a result (de Zeeuw et al., 2015).

In sum, even if it accounts for only a small proportion of variance, classroom placement appears to be associated with some twins’ behaviors in early school years. Twins also tend to be more similar when sharing the same classroom, which further supports the idea that the classroom environment may affects the twins’ behaviors. However, the findings from previous studies are mixed with regard to the specific social behaviors more likely to be affected by the classroom placement. While some studies showed significant associations with internalizing behaviors and peer problems, others did not support these patterns of findings (DiLalla & Mullineaux, 2008; Tully et al., 2004; van Leeuwen et al., 2005).

A number of limitations currently constrain existing findings. First, because the classroom placement was only assessed in the early school years, the authors often overlooked classroom placement in the subsequent years and its putative impact on the twins’ behaviors. Therefore, the association between the classroom placement (and cumulative years of classroom placement) and twins’ behaviors across the elementary school years remains largely unknown. Second, all of these studies were conducted on same-sex twin pairs and only one study controlled for the twins’ sex in their analyses. Third, one study (out of three) estimated the classroom placement on twins’ internalizing and externalizing behaviors without taking into account initial differences in these behaviors prior to school entry (Tully et al., 2004). Controlling for these initial levels is important in order to strengthen existing evidence that classroom placement is genuinely related to differences in social behaviors.

This study overcomes these limitations by statistically controlling for the sex and twins’ behaviors prior to formal schooling, and by examining whether the classroom placement is associated with twins’ behaviors at multiple occasions throughout the elementary school years. This study also extends previous findings by testing the cumulative years (i.e., total number of years) of classroom sharing on twins’ behaviors. Similar to the previous studies, we examined the association between classroom placement and several social behaviors, including prosocial behaviors, physical aggression, social withdrawal, and anxiety (DiLalla & Mullineaux, 2008; Tully et al., 2004; van Leeuwen et al., 2005). We also extended this investigation and considered inattention behaviors to test the hypothesis that twins in separate classrooms would be more inattentive than those sharing the same classroom. Lastly, we estimated the contribution of the classroom placement on the quality of the intertwin relationship. The intertwin relationship is being frequently evoked as a reason to separate twins in educational settings, but to our knowledge, this study is the first to actually test this association. Investigating the classroom placement effects on these twin’s social behaviors is of matter for the development of evidence-based educational policy for multiple birth children. It also contributes to the mission of Multiple Birth Canada by dispelling myths and by informing educationalists, psychologists and families about the effect of classroom placement on twins.

The average positive or negative association of classroom placement with twins’ behaviors, and the within-twin pairs similarity on these behaviors as a function of the classroom placement, were investigated from ages 6 to 12 years while controlling for the effects of sex and of twins’ behaviors at age 5. These associations were also examined as a function of zygosity.

## Method

### Participants

Participants were pairs of twins born in the greater Montreal area, in Quebec-Canada. They were recruited from April 1995 and December 1998 to enroll in an ongoing prospective longitudinal study of a birth cohort of twins. Parents of twins were contacted by letter and by phone. Of 989 families contacted, 662 agreed to participate (67.0%). These families were followed longitudinally from the age of 5 months onward and assessed yearly or biennially on various child and family characteristics.

Zygosity was obtained by assessment of physical similarity of twins through aggregation of independent tester ratings using the short version of the Zygosity Questionnaire for Young Twins (Goldsmith, 1991). In addition, DNA was extracted through mouth swabs collected by mothers for 31.3% of the pairs selected at random. A comparison of the two methods indicated a concordance of 92.0%. Using data available from the twins’ medical files (including data on the chorion), as well as physical similarity, there was a concordance rate of 96.0% with the data drawn from genotyping (Forget-Dubois et al., 2003). Zygosity was established for a total of 248 monozygotic (MZ) pairs and 405 dizygotic (DZ) pairs, including 196 opposite-sex pairs.

The twin’s behaviors prior to attending elementary school (Mean or M = 5.30, Standard deviation or SD = 0.27) were assessed by the mother. Teachers rated the twins’ behaviors at ages 6 (M = 6.04, SD = 0.27), 7 (M = 7.07, SD = 0.27), 9 (M = 9.08, SD = 0.29), 10 (M = 10.00, SD = 0.28), and 12 years (M = 12.09, SD = 0.28). The average attrition rate from ages 5 (*N* = 439 pairs) to 12 (*N* = 316 pairs) was 4.19% per year, although it varied slightly across measures. Twin pairs with at least one available datum on classroom placement throughout the elementary school years (*N* = 560 pairs) were included in the analyses.

### Measures and Procedure

#### Classroom placement

Teacher contact details for each twin were used from the administrative data of the study to determine if twins were in separate classrooms (coded 0) or shared the same classroom (coded 1).

#### Twins’ behaviors

Twins’ behaviors within the peer group were rated by the mothers at age 5 and by the teachers from ages 6 to 12. ***Prosocial behaviors*** were measured with three items from the Social Behavior Questionnaire (Tremblay et al., 1991). Mothers and the teachers rated how many times (0 = never; 1 = sometimes; 2 = often) in the past 12 months the child had: tried to help someone who has been hurt; comforted a child (friend, brother or sister) who was crying or upset; helped other children (friends, brother or sister) who were feeling sick. Reliability estimates were 0.81 for mother ratings and ranged from 0.84 to 0.86 for teacher ratings.

***Physical aggression*** was also rated for each twin using three items from the Social Behavior Questionnaire (Tremblay et al., 1991). Mothers and the teachers were asked how many times (0 = never; 1 = sometimes; 2 = often) in the past 12 months the child had: hit, bit, or kicked other children; physically attacked people; got into fights. Cronbach’s α for mother ratings (at age 5) was acceptable, α = 0.73. Reliability indices for teacher ratings ranged from 0.86 to 0.90.

***Social withdrawal*** was measured with three items adapted from the Revised Class Play (Masten et al., 1985). Twins were rated on a 3-point scale on how many times in the past 12 months the child had: preferred to play alone rather than with other children; tended to do things on his own, been rather solitary; sought the company of other children (reversed). The internal consistency estimates for teacher ratings (between 0.70 and 0.77) and mother ratings (α = 0.61) were acceptable.

Participants’ ***anxiety*** was rated on how often (0 = never; 1 = sometimes; 2 = often) in the past 12 months the child had: been too fearful or anxious; worried; been nervous, high-strung or tense; and cried a lot (this last item was only measured at ages 5, 6, and 7). These items were from the widely validated DSM-oriented scale for anxiety problems of the Child Behavior Checklist (CBCL) and Teacher Report Form (TRF) (Achenbach & Rescorla, 2001). This scale is reliable, with an adequate Cronbach alpha of 0.68 for mother ratings, and acceptable estimates ranging from 0.77 to 0.85 for teacher ratings.

Mothers (at age 5) and teachers (from ages 6 to 12) rated each twin’s ***inattention behaviors***, using three items adapted from the DSM-oriented scale for attention deficit/ hyperactivity problems of the CBCL or TRF (Achenbach & Rescorla, 2001). How often in the past 12 months the child had: been easily distracted, had trouble sticking to any activity; been unable to concentrate, been unable to pay attention for long; been inattentive? Reliability estimates indicate a satisfactory internal consistency of 0.89–0.91 for teacher ratings, and of 0.79 for mother ratings.

An average score of prosocial behaviors, physical aggression, social withdrawal, anxiety and inattention was computed at each age. Despite having only a few items, these measures have been extensively used in many longitudinal studies with preschool and elementary school children (Lacourse et al., 2014; Pingault et al., 2015; REF).

#### Intertwin relationship

At age 10, the quality of the intertwin relationship was measured with eight items adapted from the Network of Relationships Inventory (Furman & Buhrmester, 1985, 1992) (e.g., my twin and I always choose each other as partners for games or for work, are often angry with each other (reversed), make up easily after a fight, help each other a lot with our school work). Each twin rated these items on a 5-point scale going from 1 (not true at all) to 5 (very true). The mean score was computed for each twin across the eight items. The internal consistency of this scale is adequate (α = 0.85).

### Analytical Approach

#### Average effect of classroom placement

Multivariate regression models controlling for sex and twins’ behaviors prior to formal schooling (at age 5) were conducted to estimate the overall contribution of concurrent classroom placement on MZ and DZ twins’ prosocial behaviors, physical aggression, social withdrawal, anxiety and inattention. These models were conducted at ages 6, 7, 9, 10, and 12 years, using the classroom placement and the twins’ behaviors measured at concurrent ages, and testing for the zygosity by classroom placement interaction. A separate regression model was also performed to estimate the classroom placement on the quality of the intertwin relationship at age 10. However, since the quality of the intertwin relationship was not assessed prior to school entry, this covariate could not be included in the model.

Multivariate regression models were also conducted to test the cumulative years of classroom sharing (from ages 6 to 12) on twins’ behaviors at age 12. Once again, we controlled for the sex and twins’ behaviors prior to formal schooling and we tested the zygosity by cumulative years of classroom placement interaction. The cumulative years of classroom sharing computed from ages 6 to 10 was also tested on the quality of the intertwin relationship at age 10.

#### Similarity among twin pairs

Further multivariate regression models controlling for sex and twins’ behaviors prior to formal schooling (at age 5) were conducted on the absolute difference scores between twins of a pair to assess whether twins sharing the same classroom at ages 6, 7, 9, 10, and 12 years were more similar at concurrent ages in prosocial behaviors, physical aggression, social withdrawal, anxiety and inattention than those in separate classrooms. Smaller absolute difference scores indicate greater similarity between twins of a pair, whereas greater absolute difference scores indicate less similarity, that is, greater differences between twins of a pair. We also tested within-pair similarity using intraclass correlations.

The analyses were performed on variables corrected for age, and statistically controlling for the mother’s number of years of educational attainment and the household income. Since multiple testing were conducted, we fixed the p-value for statistical significance to .01 in all analyses. To capture the average effect of classroom placement, we used one twin selected randomly out of each pair to avoid potential bias in teacher ratings of twins’ behaviors (Lamb et al., 2012). The analyses were performed using SPSS 26.0 for Windows (SPSS Inc., Chicago, IL).

## Results

In most cases, twins were in separate classrooms during the elementary school years (68.50%, 75.60%, 76.40%, 70.50%, 60.50% at ages 6, 7, 9, 10, and 12 years, respectively). Chi-square tests of classroom placement by zygosity showed no differences between the proportion of MZ twins and the proportion of DZ twins sharing the same classroom or being in separate classrooms, across ages. About 58.0% of twins had the same classroom placement status (e.g., always together, or always separated) throughout the elementary school years, while 29.5% had gone through one transition in classroom placement status (e.g., from sharing to classroom separation, or the reverse). Only 12.5% of twins had gone through 2 or 3 transitions from one classroom placement status to another, showing overall stability in classroom placement status during the elementary school.

Means and standard deviations of twins’ behaviors and of the intertwin relationship, by classroom placement and zygosity can be found in Table 1. The mean levels of only a few behaviors significantly differed among MZ and DZ twins in same versus separate classrooms. Thus, on average, most twins’ behaviors across the elementary school years did not differ

**Table 1.** Means (*SD*) of Twins’ Behaviors by Classroom Placement and Zygosity.

Ages	Constructs	MZ		*p*	DZ		*p*
		SC	DC		SC	DC	
		*N* = 49	*N* = 112		*N* = 75	*N* = 155	
6	Prosocial behaviors	1.05 (0.55)	1.01 (0.59)	.69	1.02 (0.64)	0.85 (0.62)	.06
	Physical aggression	0.37 (0.58)	0.19 (0.40)	.02	0.35 (0.55)	0.25 (0.44)	.14
	Social withdrawal	0.51 (0.47)	0.67 (0.57)	.09	**0.52 (0.54)**	**0.81 (0.55)**	**.00**
	Anxiety	0.52 (0.60)	0.56 (0.46)	.65	0.50 (0.52)	0.52 (0.50)	.78
	Inattention	0.76 (0.71)	0.74 (0.68)	.87	0.69 (0.66)	0.83 (0.63)	.12
		*N* = 49	*N* = 131		*N* = 52	*N* = 188	
7	Prosocial behaviors	1.07 (0.55)	0.96 (0.59)	.26	0.98 (0.49)	0.93 (0.59)	.58
	Physical aggression	0.25 (0.44)	0.27 (0.50)	.81	0.32 (0.45)	0.33 (0.50)	.90
	Social withdrawal	0.70 (0.55)	0.71 (0.55)	.91	0.54 (0.53)	0.66 (0.51)	.14
	Anxiety	0.49 (0.48)	0.56 (0.52)	.41	0.58 (0.52)	0.48 (0.53)	.23
	Inattention	0.68 (0.65)	0.80 (0.70)	.30	0.92 (0.67)	0.89 (0.67)	.78
		*N* = 37	*N* = 124		*N* = 52	*N* = 167	
9	Prosocial behaviors	1.14 (0.55)	0.97 (0.62)	.14	0.96 (0.57)	0.91 (0.62)	.61
	Physical aggression	0.29 (0.52)	0.27 (0.50)	.83	0.22 (0.42)	0.25 (0.41)	.65
	Social withdrawal	0.51 (0.45)	0.58 (0.53)	.47	0.64 (0.45)	0.60 (0.53)	.62
	Anxiety	0.52 (0.55)	0.66 (0.64)	.23	0.51 (0.51)	0.61 (0.56)	.25
	Inattention	0.86 (0.79)	0.82 (0.70)	.77	0.81 (0.68)	0.86 (0.70)	.65
		*N* = 54	*N* = 111		*N* = 62	*N* = 163	
10	Prosocial behaviors	1.17 (0.51)	1.00 (0.56)	.06	1.06 (0.63)	0.94 (0.56)	.17
	Physical aggression	0.18 (0.44)	0.30 (0.48)	.12	0.22 (0.39)	0.26 (0.44)	.53
	Social withdrawal	**0.27 (0.39)**	**0.65 (0.56)**	**.00**	0.39 (0.45)	0.55 (0.53)	.04
	Anxiety	0.48 (0.45)	0.61 (0.55)	.13	0.45 (0.56)	0.50 (0.49)	.51
	Inattention	0.77 (0.70)	0.83 (0.69)	.60	0.76 (0.73)	0.78 (0.71)	.85
	Intertwin relationship	3.30 (0.90)	3.24 (1.01)	.68	3.29 (0.92)	3.10 (0.96)	.19
		*N* = 50	*N* = 70		*N* = 76	*N* = 121	
12	Prosocial behaviors	**1.14 (0.61)**	**0.85 (0.61)**	**.01**	1.02 (0.59)	0.88 (0.63)	.12
	Physical aggression	0.07 (0.24)	0.23 (0.45)	.02	0.14 (0.34)	0.15 (0.33)	.84
	Social withdrawal	0.49 (0.49)	0.51 (0.55)	.84	0.57 (0.53)	0.60 (0.55)	.71
	Anxiety	0.42 (0.48)	0.43 (0.49)	.91	0.44 (0.46)	0.52 (0.57)	.30
	Inattention	0.52 (0.63)	0.69 (0.70)	.17	0.51 (0.66)	0.73 (0.74)	.04

*Note*. One twin selected randomly. MZ = monozygotic twins; DZ = dizygotic twins; SC = same classroom; DC = different classrooms. Bold indicates significance at *p* < .01.

according to classroom placement.

### Is Classroom Placement Associated with Twins’ Behaviors and the Intertwin Relationship?

Results from the multivariate linear regressions are shown in Table 2. At age 6, classroom sharing was significantly associated with lower levels of twins’ social withdrawal (β = -0.19, *p* < .001, *r*^2^ = 0.036) after controlling for sex and initial differences at age 5 (baseline level, see Table 2). Classroom placement did not significantly predict physical aggression, prosocial behaviors, anxiety and inattention behaviors at age 6, nor any of the twins’ behaviors at ages 7 and 9 years. At age 10, twins sharing the same classroom were less socially withdrawn compared to twins in separate classrooms (β = −0.20, *p* < .001, *r*^2^ = 0.040). Classroom placement was not significantly associated with any other twins’ behaviors or with the quality of the intertwin relationship. At age 12, classroom sharing was significantly associated with lower levels of physical aggression (β = −0.17, *p* = .004, *r*^2^ = 0.029) and inattention (β = -0.18, *p* = .003, *r*^2^ = .033). Classroom placement at age 12 was not significantly associated with any other twins’ behaviors. Overall, classroom placement explained small (between 2.9% and 4.0%) but significant proportion of the variance in some social behaviors across the elementary school years. No significant interaction between zygosity and classroom placement were found in the prediction of twins’ behaviors across the different ages or the intertwin relationship at age 10.

**Table 2.** Linear Regressions Predicting Twins’ Behaviors from being in the Same or Different Classrooms.

Ages	Constructs	Model A				Model B
		Sex	Zygosity	Baseline	SC/DC	SC/DC X zygosity
6	Prosocial behaviors	0.11	−0.05	0.05	0.08	−0.01
	Physical aggression	−0.18**	0.07	0.14	0.13	0.04
	Social withdrawal	0.00	0.07	0.15**	−0.19***	−0.24
	Anxiety	0.07	−0.02	0.07	−0.04	0.02
	Inattention	−0.20***	0.03	0.30***	−0.04	−0.10
7	Prosocial behaviors	0.23***	−0.05	0.05	0.04	−0.10
	Physical aggression	−0.34***	0.02	0.28***	0.00	0.13
	Social withdrawal	−0.19***	−0.13	0.25***	−0.07	−0.25
	Anxiety	−0.05	−0.06	0.16**	0.02	0.15
	Inattention	−0.17**	0.09	0.23***	−0.01	0.13
9	Prosocial behaviors	0.25***	−0.02	0.04	0.08	−0.01
	Physical aggression	−0.26***	−0.05	0.12	0.02	−0.19
	Social withdrawal	−0.10	0.07	0.03	−0.03	0.15
	Anxiety	0.01	−0.07	−0.02	−0.06	−0.04
	Inattention	−0.16**	1.03	0.21***	0.06	−0.10
10	Prosocial behaviors	0.16**	−0.03	0.12	0.05	−0.10
	Physical aggression	−0.34***	−0.03	0.13	−0.09	0.03
	Social withdrawal	−0.09	−0.05	0.12	−0.20***	0.28
	Anxiety	−0.04	−0.08	−0.02	−0.09	0.11
	Inattention	−0.16	0.00	0.26***	−0.01	0.13
	Intertwin relationship	0.18***	−0.05	–	0.06	0.16
12	Prosocial behaviors	0.30***	0.06	0.17**	0.13	−0.30
	Physical aggression	−0.34***	−0.13	0.11	−0.17**	0.07
	Social withdrawal	−0.11	0.03	0.22	−0.05	−0.05
	Anxiety	−0.01	0.06	0.02	−0.06	−0.17
	Inattention	−0.21***	−0.03	0.20**	−0.18**	−0.03

*Note*. All parameters are standardized beta coefficients. One twin selected randomly.

Model A: prediction of twins’ behaviors from sex, zygosity, baseline (the outcome behavior at age 5), and SC/DC (twins being in the same or different classrooms at concurrent year of outcome).

Model B: prediction of twins’ behaviors from the same four covariates as model A and the interaction term between SC/DC and zygosity. The classroom placement explained between 2.9% and 4.0% of variance in twins’ behaviors.

** *p* < .01. ***p < .001.

#### Cumulative years of classroom sharing

Associations between the number of years spent in the same classroom and twins’ behaviors at age 12 are shown in Table 3. Cumulative years of classroom sharing significantly predicted physical aggression at age 12, after controlling for sex and levels of physical aggression at age 5. The more years twins shared the same classroom, the less they displayed physical aggression (β = -0.16, *p* = .009, *r*^2^ = 0.024). Cumulative years of classroom sharing did not predict twins’ prosocial behaviors, social withdrawal, anxiety or inattention at age 12. Similarly, no significant association was found between the number of years of classroom sharing and the quality of the intertwin relationship at age 10. No significant interaction between zygosity and cumulative years of classroom placement were found in the prediction of twins’ behaviors, or the intertwin relationship.

**Table 3.** Linear Regressions Predicting Twins’ Behaviors from Number of Years in the Same Classroom.

Age	Constructs	Model A				Model B
		Sex	Zygosity	Baseline	nSC/DC	nSC/DC X zygosity
10	Intertwin relationship	0.18***	−0.06	–	0.03	0.19
12	Prosocial behaviors	0.29***	0.05	0.18**	0.06	−0.51
	Physical aggression	0.33***	−0.12	0.12	−0.16**	0.25
	Social withdrawal	−0.11	0.03	0.22***	−0.02	−0.09
	Anxiety	−0.01	0.07	0.01	0.02	−0.14
	Inattention	−0.20**	−0.02	0.21***	−0.05	−0.01

*Note*. All parameters are standardized beta coefficients. One twin selected randomly.

Model A: prediction of twins’ behaviors from sex, zygosity, baseline (the outcome behavior at age 5), nSC/DC (number of years spent in the same classroom).

Model B: prediction of twins’ behaviors from the same four covariates as model A and the interaction term between nSC/DC and zygosity. Cumulative years of classroom sharing explained 2.4% of the physical aggression variance at age 12.

** *p* < .01. ***p < .001.

### Do Twins Behave More Similarly When Sharing the Same Classroom?

Results from the multivariate linear regressions conducted on the absolute within-pair differences for each social behavior are shown in Table 4. Classroom sharing was significantly associated with less difference (i.e., higher twin similarity) in anxiety (β = -0.24, *p* < .001, *r*^2^ = 0.059) and inattention (β = -0.18, *p* = .002, *r*^2^ = 0.031) at age 6, in prosocial behaviors at ages 7 (β = -0.23, *p* < .001, *r*^2^ = 0.052), 9 (β = -0.18, *p* = .004, *r*^2^ = 0.030), and 10 (β = -0.17, *p* = .005, *r*^2^ = 0.027), and in social withdrawal at age 12 (β = -0.22, *p* < .001, *r*^2^ = 0.050). Classroom placement therefore explained a small but significant portion of variance (between 2.7% and 5.9%) in twins’ similarity. This conclusion was also supported by the examination of MZ and DZ intraclass correlations, showing apparent higher magnitude of intraclass correlations (i.e., greater similarity) for twins sharing the same classroom (see Table S1 in the online supplemental material). No significant zygosity by classroom placement interactions were found.

**Table 4.** Linear Regressions Predicting Absolute Difference in Twins’ Behaviors from being in the Same or Different Classrooms.

Ages	Constructs	Model A				Model B
		Sex	Zygosity	Baseline	SC/DC	SC/DC X zygosity
6	Prosocial behaviors	−0.02	0.10	−0.05	−0.14	−0.09
	Physical aggression	−0.14	0.20***	0.08	0.06	0.28
	Social withdrawal	0.02	0.06	0.06	−0.12	−0.02
	Anxiety	−0.02	0.04	0.07	−0.24***	0.02
	Inattention	−0.04	0.11	0.08	−0.18**	0.11
7	Prosocial behaviors	0.09	0.05	0.06	−0.23***	−0.02
	Physical aggression	−0.25***	0.10	0.05	0.01	−0.06
	Social withdrawal	−0.07	−0.08	0.12	−0.03	0.11
	Anxiety	−0.02	0.08	0.05	−0.08	0.22
	Inattention	−0.13	0.05	0.03	−0.12	−0.12
9	Prosocial behaviors	−0.06	0.14	−0.01	−0.18**	−0.19
	Physical aggression	−0.12	0.08	0.02	−0.07	0.05
	Social withdrawal	−0.12	0.14	0.02	0.01	0.17
	Anxiety	−0.01	0.07	−0.02	−0.15	0.26
	Inattention	0.03	0.17**	0.13	−0.05	0.08
10	Prosocial behaviors	−0.09	0.10	0.16**	−0.17**	0.31
	Physical aggression	−0.29***	0.08	0.02	−0.11	−0.11
	Social withdrawal	−0.08	0.04	0.08	−0.07	0.17
	Anxiety	0.03	0.10	0.04	−0.10	0.24
	Inattention	−0.10	0.22***	−0.05	−0.11	0.31
	Intertwin relationship	−0.08	0.14	–	−0.09	0.15
12	Prosocial behaviors	−0.10	0.08	0.01	−0.19	0.20
	Physical aggression	−0.25***	0.07	0.11	−0.10	0.08
	Social withdrawal	−0.15	−0.01	0.09	−0.22***	−0.21
	Anxiety	−0.06	0.19**	0.02	−0.12	−0.03
	Inattention	−0.04	0.17	−0.00	−0.09	−0.02

*Note*. All parameters are standardized beta coefficients.

Model A: prediction of absolute difference scores between twins of a pair on each behavior from sex, zygosity, baseline (absolute difference score between twins of a pair on the outcome behavior at age 5), and SC/DC (twins being in the same or different classrooms at concurrent year of outcome).

Model B: prediction of absolute difference scores of twins’ behaviors from the same four covariates as model A and the interaction term between SC/DC and zygosity. The classroom placement explained between 2.7% and 5.9% of variance in twin similarity on these behaviors.

** *p* < .01. ***p < .001.

## Discussion

The purpose of this study was twofold. First, the goal was to examine whether the classroom placement of twins (i.e., assignment to the same versus different classrooms) was positively or negatively associated with their prosocial behaviors, physical aggression, social withdrawal, anxiety and inattention across the elementary school years (i.e., 6 to 12 years), as well as their intertwin relationship at age 10. Second, the study aimed to estimate the within-pair similarity in regard to these behaviors as a function of the classroom placement. Our study revealed an association of small magnitude between classroom placement, and cumulative years of classroom placement, with twins’ behaviors. Twins sharing the same classroom were less socially withdrawn at ages 6 and 10 and displayed lower levels of physical aggression and inattentive behaviors at age 12. The more years twins shared the same classroom, the less physically aggressive they were at age 12. Moreover, twin pairs sharing the same classroom were overall, slightly more similar on their behaviors than twins in separate classrooms, but this association reached statistical significance only for anxiety and inattention at age 6, prosocial behaviors from ages 7 to 10, and social withdrawal at age 12.

These findings provide evidence that educating twins together is associated with modest positive outcomes in twins’ behaviors and social functioning at school. This study is also the first to show that classroom sharing at age 12 is significantly associated with lower levels of inattention behaviors at concurrent age, which gives strength to the hypothesis that classroom separation could create a distraction for the twins at school.

These results parallel previous studies that found small, but significant associations between classroom placement and twins’ less frequent social behavioral problems at the beginning of the elementary school years. Similar to van Leeuwen et al. (2005), we found that classroom sharing was associated with lower levels of social withdrawal at age 6. Like most studies, our findings also showed no association between classroom placement in early school years and prosocial behaviors and externalizing behaviors, such as conduct problems and physical aggression at ages 6 or 7 (DiLalla & Mullineaux, 2008; Tully et al., 2004; van Leeuwen et al., 2005). However, while previous studies reported higher levels of twins’ internalizing behaviors (including anxiety) when placed in different classrooms (Tully et al., 2004; van Leeuwen et al., 2005), we found no such association in our study. Our study did not either support the previously reported association between classroom separation during early grades and higher levels of twins’ anxiety, above and beyond the anxiety or stress experienced by most children when starting formal schooling (Parent et al., 2019).

This absence of significant association with internalizing difficulties could be explained by the context in which the decisions for classroom sharing/separation are made. For instance, decision making for classroom placement is more likely to stem from state and/or district-level policy in the US. By recommending leaving the final decision at the family level (parents and children themselves), decisions for classroom placement in Canada are more likely to reflect the wishes of families and children (Staton et al., 2012). This could explain why no association with internalizing difficulties were found in our study, in comparison to findings from other countries. Another potential explanation for these inconsistencies is that, unlike Tully et al. (2004), we controlled for these specific behaviors prior to formal schooling. Differences with previous studies could also be due to methodological aspects (e.g., use of an aggregate score of internalizing behaviors), the time of measurement (e.g., assessing classroom placement at age 5 versus classroom placement at age 6), and the fact that different informants rated the twins’ behaviors prior to school entry (i.e., mother ratings at age 5) and subsequently (i.e., teacher ratings from ages 6 to 12), unlike van Leeuwen et al. (2005). Other factors at the individual-, classroom- or school-level may also buffer the contribution of classroom placement on anxiety (Hamre & Pianta, 2005; Kwon et al., 2019; Wang et al., 2020). It has been hypothesized that impact of classroom placement depends on individual characteristics and different perceptions of the classroom experience. For example, one study found that MZ twins experience differently peer and academic stressors within the classroom, as well as the relationship with the teacher (Asbury et al., 2008). Individual characteristics such as the temperamental style and individual-specific life-events, such as birth complications, disability, missing school due to illness, and peer-relations, could also account for variations among twin pairs and lead to different experience of classroom placement. Teachers and classmates may also treat twins in the same classroom differently, responding to their individual characteristics. In turn, factors such as the classroom climate (Wang et al., 2020) and the teacher-student relationship (Hamre & Pianta, 2005) are associated with twins’ behaviors at school. Our study did not include measured factors at the individual or classroom-level. Therefore, we could not test for moderation effects of specific individual (e.g., temperament) or classroom factors (e.g., classroom climate) in the association between classroom placement and twins’ social behaviors, not we could control for these potential confounders.

Sharing a classroom has previously been hypothesized to be a potential limitation for twins’ social identity and interaction/behaviors toward peers (Alexander, 2012; DiLalla, 2006; Hay & Preedy, 2006; Lalonde & Moisan, 2003; Staton et al., 2012). Interestingly, our study did not support this hypothesis as we found no significant effect of classroom placement on prosocial behaviors. However, we observed lower physical aggression when sharing the same classroom at age 12. Despite the modest effect size, our results rather show that twins are less physically aggressive when educated together, and this seems reinforcing for twins sharing the same classroom over the elementary school years. Twins’ self-esteem and self-confidence might be greater in the presence of a co-twin, which has been previously associated with lower physical aggression (Diamantopoulou et al., 2008; Lee 2014; Sandstrom & Jordan, 2008). It is also possible that twins’ similarity on their prosocial behaviors and social withdrawal when sharing the same classroom evoke similar positive and reinforcing reactions from peers (Segal et al., 2018), leading to lower levels of physical aggression. However, another explanation is that separation of children may occur in response to emergence of aggressive behaviors. Preschool hyperactivity/impulsivity, inattention, and oppositional behaviors has been suggested to be the greatest driving force behind the decision to separate the twins in kindergarten and afterward (Coventry et al., 2009). Despite the fact that we controlled for prior-to-school behaviors, physical aggression might have only emerged at school entry or be precipitated by other contextual/family factors during formal schooling. Classroom separation may be a common response to addressing issues related to aggressive behaviors from twins. This would explain why twins sharing the same classroom had lower levels of physical aggression. Future studies should further explore this hypothesis by disentangling the pattern of association between classroom separation and behavioral difficulties over time, using cross-lagged modeling.

Surprisingly, classroom placement or the cumulative years of classroom placement did not predict the quality of the intertwin relationship at age 10 or the similarity of twins’ perceptions of the intertwin relationship. In other words, the shared (versus non-shared) experience derived from the classroom placement does not seem to be related to the quality of the twins’ relationship with each other. Therefore, the present results do not support the notion that classroom separation of twins benefits the intertwin relationship. This result should, however, be interpreted with caution as the intertwin relationship was only measured at one single point in time. Future studies are needed to further explore how the within-twin pair relationship maps onto decision-making for classroom placement. One should also keep in mind that, even if we found no significant average classroom sharing effects on most child’s outcomes, these results do not translate into individual effects of classroom placement. Classroom sharing (and factors at the classroom-level) may have a profound social influence, positive or negative, on an individual student that is not captured at the classroom mean-level.

### Limitations

Our study is not without limitations. First, it should be acknowledged that we did not explicitly assess classroom-level factors such as the classroom climate or teacher characteristics known to be associated with children’s social development (Hamre & Pianta, 2005; Kwon et al., 2019; Wang et al., 2020). We also did not control for broader school-level factors or the frequency of twins’ interactions outside the classroom such as sharing recess and lunch at school. Second, it should also be noted that this study is a not a randomized controlled trial of twin’s classroom placement, which prevents causal interpretation. Children might not be randomly placed in a same or different classroom, leading to potential additional family-wide factors to be taken into account for future studies of classroom placement. The average classroom placement effect on twins’ social behaviors and twins’ similarity on these behaviors was, however, significant after controlling for the mother’s educational attainment and the household income. Third, different informants reported twin’s behaviors prior to formal schooling (maternal assessment) and during the elementary school years (teacher assessment). Moreover, because twins’ behaviors at school (ages 6 to 12) were reported by the teacher, twin pairs sharing the same classroom were rated by the same informant. It is possible that teachers of twins in the same classroom rated twins more similarly than teachers of twins in separate classrooms. While this potential bias was avoided by selecting one twin out of each pair to estimate the average positive or negative associations of classroom placement with twins’ behaviors, it could have been a limitation to estimate the within-pair similarity as a function of classroom placement.

Despite these few limitations, our study strengthens findings from previous research that showed slightly greater similarity among twins sharing the same classroom, and a small but significant average association (2%–4% of variance) between the classroom environment and children’s social, externalizing, and internalizing behaviors (DiLalla & Mullineaux, 2008; Tully et al., 2004; Wang et al., 2020). This study also added to previous literature by investigating the classroom placement effects on the twin’s inattention behaviors and on the intertwin relationship.

### Implication for Educational Policy

These results have practical implications for guiding decision-making of parents and teachers, and for orienting policy in educational settings. Classroom sharing during elementary school was associated with small improvement of some twins’ social behaviors, especially when transitioning into early adolescence. However, despite the positive behavioral outcomes associated with classroom sharing of twins, this study revealed that classroom placement did not predict most twins’ behaviors during the elementary school years. These results highlight that organizational-level policy (i.e., school, district or state) about classroom placement of twins is unjustified and rather suggests that a one-size-fits-all approach to classroom placement of twins is not optimal. Therefore, schools should support an individual-level approach for decision making in favor of parents’ and twins’ individual decision and needs.

## Supplemental Material

sj-pdf-1-epx-10.1177_08959048211015626 – Supplemental material for Classroom Placement and Twins’ Social Behaviors in Elementary School: Providing Empirical Evidence to Inform Educational Policy Click here for additional data file. Supplemental material, sj-pdf-1-epx-10.1177_08959048211015626 for Classroom Placement and Twins’ Social Behaviors in Elementary School: Providing Empirical Evidence to Inform Educational Policy by Gabrielle Garon-Carrier, Vincent Bégin, Mara Brendgen, Frank Vitaro, Isabelle Ouellet-Morin, Ginette Dionne and Michel Boivin in Educational Policy
