# Supplementary material for: Classroom Placement and Twins’ Social Behaviors in Elementary School: Providing Empirical Evidence to Inform Educational Policy
Source: Educ Policy (Los Altos Calif). 2021 May 27;36(7):1850–75. doi: 10.1177/08959048211015626 (PMC9493409; doi:10.1177/08959048211015626)
Supplement: sj-pdf-1-epx-10.1177_08959048211015626 – Supplemental material for Classroom Placement and Twins’ Social Behaviors in Elementary School: Providing Empirical Evidence to Inform Educational Policy [file sj-pdf-1-epx-10.1177_08959048211015626.pdf]

**Table S1.** Intra-class correlations coefficients by classroom placement and zygosity

| Age | Construct | MZ | | DZ | |
|---|---|---|---|---|---|
| | | **SC (n = 45)** | **DC (n = 104)** | **SC (n = 68)** | **DC (n = 140)** |
| 6 | Prosocial | .583 (.352 – .747) | .353 (.173 – .511) | .558 (.370 – .702) | .191 (.026 – .346) |
| | Anxiety | .700 (.513 – .823) | .134 (-.060 – .317) | .546 (.355 – .693) | .151 (-.015 – .309) |
| | Inattention | .732 (.560 – .843) | .429 (.259 – .574) | .310 (.079 – .509) | .166 (.001 – .323) |
| | Social withdrawal | .437 (.167 – .645) | .388 (.212 – .540) | .244 (.006 – .456) | .178 (.013 – .334) |
| | Physical aggressivity | .822 (.699 – .898) | .617 (.482 – .723) | .308 (.077 – .508) | .220 (.056 – .372) |
| **Age** | **Construct** | **SC (n = 47)** | **DC (n = 123)** | **SC (n = 51)** | **DC (n = 177)** |
| 7 | Prosocial | .674 (.482 – .804) | .272 (.099 – .429) | .556 (.333 – .720) | .167 (.019 – .308) |
| | Anxiety | .493 (.242 – .682) | .216 (.041 – .379) | .118 (-.161 – .379) | .119 (-.028 – .262) |
| | Inattention | .679 (.489 – .807) | .575 (.443 – .682) | .496 (.257 – .677) | .356 (.221 – .478) |
| | Social withdrawal | .387 (.115 – .605) | .218 (.043 – .379) | .186 (-.092 – .437) | .178 (.032 – .317) |
| | Physical aggressivity | .516 (.271 – .698) | .643 (.527 – .736) | .322 (.054 – .547) | .101 (-.047 – .245) |
| **Age** | **Construct** | **SC (n = 37)** | **DC (n = 113)** | **SC (n = 50)** | **DC (n = 156)** |
| 9 | Prosocial | .675 (.452 – .818) | .340 (.164 – .494) | .523 (.288 – .698) | .109 (-.050 – .262) |
| | Anxiety | .737 (.546 – .855) | .350 (.177 – .502) | .205 (-.075 – .455) | .001 (-.156 – .158) |
| | Inattention | .675 (.452 – .818) | .571 (.432 – .683) | .222 (-.057 – .469) | .167 (.010 – .315) |
| | Social withdrawal | .469 (.175 – .686) | .488 (.334 – .617) | .133 (-.148 – .395) | .266 (.114 – .406) |
| | Physical aggressivity | .792 (.632 – .887) | .490 (.336 – .618) | .082 (-.198 – .350) | .191 (.035 – .337) |
| **Age** | **Construct** | **SC (n = 54)** | **DC (n = 103)** | **SC (n = 61)** | **DC (n = 153)** |
| 10 | Prosocial | .682 (.508 – .802) | .154 (-.042 – .338) | .309 (.063 – .519) | .242 (.086 – .386) |
| | Anxiety | .584 (.376 – .735) | .259 (.070 – .431) | .139 (-.115 – .376) | .064 (-.095 – .221) |
| | Inattention | .792 (.667 – .874) | .396 (.220 – .547) | .181 (-.072 – .413) | .144 (-.015 – .295) |
| | Social withdrawal | .492 (.261 – .670) | .422 (.250 – .569) | .189 (-.064 – .420) | .210 (.054 – .357) |
| | Physical aggressivity | .742 (.593 – .842) | .672 (.551 – .766) | .402 (.170 – .593) | .207 (.051 – .354) |
| | Intertwin relationship | .754 (.606 – .852) | .673 (.550 – .767) | .621 (.431 – .758) | .499 (.366 – .613) |
| **Age** | **Construct** | **SC (n = 48)** | **DC (n = 61)** | **SC (n = 70)** | **DC (n = 103)** |
| 12 | Prosocial behaviors | .785 (.645 – .874) | .305 (.060 – .516) | .371 (.151 – .557) | .161 (-.033 – .343) |
| | Anxiety | .620 (.410 – .767) | .406 (.174 – .596) | .326 (.100 – .520) | .123 (-.072 – .308) |
| | Inattention | .722 (.533 – .834) | .418 (.188 – .605) | .314 (.086 – .510) | .29 (.103 – .459) |
| | Social withdrawal | .627 (.419 – .772) | .271 (.023 – .488) | .479 (.276 – .640) | .254 (.064 – .426) |
| | Physical aggression | .648 (.448 – .786) | .557 (.357 – .708) | .171 (-.065 – .389) | .225 (.034 – .400) |

All intra-class correlations are two-way mixed effects models. Single measure coefficients are reported. 95 % confidence intervals are reported in parentheses.
MZ: monozygotic twins, DZ: dizygotic twins, SC: same classroom, DC: different classrooms.
